# Advancing ecological validity and clinical utility in virtual reality-based continuous performance test: exploring the effects of task difficulty and environmental distractors

**DOI:** 10.3389/fpsyt.2023.1329221

**Published:** 2024-01-17

**Authors:** Hyang-Kyeong Oh, Yoon Jae Cho, Jae-Jin Kim, Bokyoung Shin, Soo-Jeong Kim, Soobin Park, Jeong-Ho Seok, Soyeon Kim, Eunjoo Kim

**Affiliations:** ^1^Institute of Behavioral Sciences in Medicine, Yonsei University College of Medicine, Seoul, Republic of Korea; ^2^Department of Psychiatry, Gangnam Severance Hospital, Yonsei University College of Medicine, Seoul, Republic of Korea; ^3^Department of Psychiatry, Bundang Jesaeng General Hospital, Seoul, Republic of Korea

**Keywords:** continuous performance test (CPT), virtual reality, ecological validity, environmental distractor, electroencephalograms (EEGs)

## Abstract

**Introduction:**

Researchers have highlighted concerns regarding the limited diagnostic utility and ecological validity of the Continuous Performance Test (CPT). Recent advancements in VR-based CPTs have attempted to address these concerns by simulating real-life scenarios and enhancing attention deficit hyperactivity disorder (ADHD) diagnosis; however, certain areas require improvement for obtaining reliable data from both healthy individuals and those with ADHD. To tackle these issues, we developed an enhanced VR-based CPT program featuring four distinct difficulty levels, advancing toward home-based assessment.

**Method:**

Our feasibility study involved subjects without ADHD to establish a normative profile for VR-based CPT before extending it to ADHD assessment. Our sample included 20 Korean adults. They received a VR device with the VR-based CPT program installed and were asked to perform 1-2 blocks per day at home. Participants were instructed to complete 12 blocks over the subsequent 2 weeks. Psychological assessments and electroencephalograms (EEGs) were administered before and after the program. Post-study usability measures were also collected.

**Result:**

Higher commission errors were notably evident in the “very high” difficulty level which featured complex stimuli and increased distraction. A notable correlation emerged between the overall distraction level and CPT accuracy, along with a significant link between intensity scores and commission errors. No significant differences were found in psychological assessment and there were no significant changes in the Theta-Beta Ratio (TBR) index before and after the program. The usability of our program was fair.

**Discussion:**

The study reveals that the newly designed VR-CPT program, simulating diverse real-life environments and offering varying task difficulty levels, proved acceptable and feasible. The key point of our study was that the adjustment and segmentation of difficulty levels in the VR-based CPT were achieved, and that this effort was validated by examining the impact of different levels of difficulty on CPT measures. Implementing this experimental setup in a home-based environment increased ecological validity, as well as clinical utility. Limitations and suggested directions for further investigation are described in detail.

## 1 Introduction

The continuous performance test (CPT) is a widely used computer-based neuropsychological approach for assessing individual's attention (1). Although the CPT paradigm may vary greatly based on the way cognitive demands are placed and task parameters are manipulated, CPTs usually require participants to remain vigilant to a specific stimulus in a continuous stream of distractors for approximately 10–30 min (2, 3). The test yields metrics such as correct hits, omission errors (OE), commission errors (CE), and reaction time variability (RTV), providing insights into an individual's attentional vulnerabilities. CPTs are frequently used to assess attention deficit hyperactivity disorder (ADHD) and aid its diagnosis (1). CPTs are objective tests that can be used to support clinical decisions, overcoming the limitations of questionnaire-based instruments including subjectivity and superficiality.

Despite its widespread use, researchers have raised concerns regarding the limited diagnostic utility of CPTs (4, 5). Past studies have yielded mixed results regarding the discriminatory ability of the CPT between individuals with ADHD and controls without ADHD. Additionally, other studies have identified a weak association between clinically reported attentional symptoms and CPT performance (6–8). While some CPTs can differentiate individuals with attention problems from healthy controls, some reports indicate that the sensitivity and specificity of the CPT as an assessment tool are < 70% (6, 9). Thus, achieving precise CPT-based single-subject classification in real clinical settings remains a challenge (4, 10).

There are several possible explanations for the low diagnostic utility of the CPT. First, an individual's attention performance in the CPT is influenced by instability and fluctuations. Intra-individual variability (IIV) has been observed repeatedly in individuals with ADHD and is known to reflect instability in information processing within the brain (11). IIV is a ubiquitous and general characteristic feature of ADHD that contributes to the observed attention and cognitive heterogeneity in individuals with ADHD (12). Studies have reported IIV not only over a period of seconds or milliseconds within a single task but also over longer periods spanning days. Inconsistency in performance is influenced by various factors such as the individual's current state, environmental stress, etc., which can affect attention processing (13, 14). As a result, the pattern of performance in CPTs can differ greatly trial-by-trial or day-by-day, with individuals scoring in the normal range on one day and showing underperformance in another. Moreover, in the case of children and adolescents, maturation during the developmental phase leads to inconsistencies in long-term performance. Some researchers have suggested that CPT sensitivity in older adolescents is lower than that in younger children (15–17). This may be because differences in attention span between individuals with ADHD and healthy controls becomes so subtle in late adolescence that they are often undetectable using the traditional CPT. Therefore, while IIV itself does serve as a behavioral marker of attention deficit in ADHD, a single-session assessment may be insufficient to reliably detect and assess attention problems.

Second, the ecological validity of most CPT implementations is low. Traditional CPTs are usually conducted using computer screens in controlled laboratory environments. This approach has the advantage of consistent and reproducible test presentation; however, the question remains of how closely such a task can reliably mimic the challenges individuals encounter in everyday life, where environments are substantially more complex (4, 18). In reality, people are constantly exposed to various distracting stimuli, such as noise, clutter, and interruptions; therefore, their distractibility is bound to be much higher than that in controlled laboratory settings. Barkley stressed the necessity of enhancing the ecological relevance of CPTs by assessing attentional abilities in realistic contexts (19). Furthermore, recent meta-analyses have highlighted the importance of focusing not only on task performance but also on the demands of the surrounding environment, as they influence distractibility and, by extension, exacerbate attention deficits (20).

In recent times, more ecologically valid CPTs have been developed through technological advancements, particularly in virtual reality (VR). VR-based CPT programs immerse users in real-life scenarios while they perform traditional CPT tasks, during which administrators can provide precise control over distracting stimuli (21, 22). This application integrates distracting stimuli as environmental demands, mirroring real-life challenges and enhancing the ecological validity of assessments. There are two different VR-based programs designed for children and adolescents with ADHD: The Virtual Classroom (VC) by Rizzo et al. (21) and the AULA Nesplora by Iriarte et al. (22), both designed for children and adolescents with ADHD. Both programs present a virtual classroom environment, and users are required to perform the CPT on a virtual blackboard (21, 22). During the CPT, various visual, auditory, and audiovisual distractors appear and disappear. Utilizing typical classroom distractions (e.g., dropping pencils and moving chairs), they aimed to maximize the parallels between a real-world classroom and the virtual environment (23, 24). Numerous subsequent studies have found that individuals with ADHD are more affected by the insertion of distractors than controls without ADHD. Moreover, these VR-based CPTs have demonstrated their potential utility, showing improved ability to distinguish between ADHD and non-ADHD groups compared to traditional CPTs, while enhancing ecological validity (25–28).

Although significant group differences between ADHD patients and healthy controls were identified in previous VR-based CPT programs, information about the normal profile regarding the influence of distraction in VR-based CPT is unclear, with mixed results obtained for its impact on CPT performance in controls without ADHD. For example, Parsons et al. (26) found distractor-induced increases in error rates (26), whereas Negut et al. (29) did not. More recently, Wiebe et al. (4) developed the first VR-based CPT program tailored for adults, called the Virtual Seminar Room (VSR). The VSR is similar to the VC paradigms but with a longer duration of 48 min and higher number of phases (eight repeated alterations between distractor-present and distractor-absent task phases) to create a more cognitively demanding and sensitive program (4). A feasibility study for controls without ADHD failed to find performance differences between the distractor-present and distractor-absent conditions. Considering the raw data, which indicated that participants made only a few errors throughout the entire task, the researchers concluded that the VSR program was still not sufficiently sensitive to detect minor performance declines (4, 21, 29). Because this was possibly due to a ceiling effect, the authors suggested increasing the task difficulty of VR-based CPT for future studies (4). As such, whether the effect of distraction effect is ubiquitous to individuals with and without ADHD and how distractors affect attention in those without ADHD remains controversial. This calls for improvement in the design of task paradigms to understand how distractors affect attentional processes in individuals without ADHD and to comprehend the pathological features of attention. Obtaining accurate information on normative standards, preceded by clinical profiles, is particularly crucial for assessment tools. This not only enhances our insight regarding distractibility in individuals without ADHD but also serves as fundamental evidence to determine and quantify attention deficits in those with ADHD.

We developed “Pay Attention!” as an improved and complementary version of an existing VR-based CPT. Our team produced four diverse and familiar real-life scenarios (room, library, outdoors, and café) and recruited adults from a wide age group without specifying a specific age band. As a solution to the task difficulty problem, we incorporated four distinct difficulty levels in each of the four locations, ranging from low to very high. Different difficulty levels varied in the level of distraction, complexity of target and non-target stimuli, and inter-stimulus intervals of the CPT. To the best of our knowledge, all previous VR-based CPTs have presented only two dichotomous conditions: distractor present and distractor absent (4, 21, 22). Since a distractor-free state is very rare in real life, a comparison between these two conditions is not ecologically valid. In future applications of this tool, administrators may apply the version with the difficulty level appropriate to the subject so that it is equally challenging to all, which may help avoid the floor and ceiling effect of the traditional CPT (4). Through the design of a wider range of experimental conditions, we can gain a better understanding of how adults with and without attention problems respond to distractions of varying levels in close-to-real-life environments. This moves us beyond merely determining whether differences exist and may allow us to obtain more statistically and ecologically plausible data that reflect the actual real-life performance of individual. Furthermore, to minimize the impact of IIV, our version of the VR-based CPT comprised multiple similar tasks within each difficulty level and was administered over an extended period rather than in a single session (12). “Pay Attention!” is also the first step toward home-based use of VR-based CPTs. Through this approach, we expect to collect ecologically valid data in a more naturalistic setting compared to conventional CPTs, where participants are more likely to be comfortable and exhibit their typical performance (30). Conducting the test at home also allowed for ongoing data collection without requiring the participants to visit a research facility.

With the above novel innovations, we aim to make VR-based CPT a more cost-effective and accessible test to a wider range of individuals who require assessment and connection to treatment for their attention problems (31, 32). We conducted a feasibility study involving subjects without ADHD, hoping to establish a normative CPT profile and broaden our understanding before applying it to an ADHD group. Additionally, we examined the impact of different levels of distraction on CPT performance within a virtual environment. Administered over several days in a home-based setting, we also evaluated whether psychological symptoms known to affect attention impairment (depression, anxiety, and stress level) changed before and after participating in the study, and whether these changes were related to CPT performance (33–35). Furthermore, changes in the theta-beta ratio (TBR) among collected electroencephalography (EEG) parameters, known to be associated with attentional control, were also measured to complement the behavioral parameters and examine neural markers related to distractibility.

## 2 Materials and methods

### 2.1 Participants

Our sample included 20 Korean adults recruited through online advertisements. The inclusion criteria were as follows: (1) age range of 19–60 years, (2) literacy to read and understand consent, and (3) absence of medical and psychiatric illness. The exclusion criteria were as follows: (1) illiteracy or inability to read the consent and understand our research process, evaluated by level of education (bachelor's degree or equivalent); (2) current use of psychotropic medication or a history of substance use disorder; (3) past or current diagnosis of a serious neurological or medical disorder; and (4) any psychiatric disorder, including baseline suicidality. To verify these criteria, The Mini-International Neuropsychiatric Interview (MINI) (36) was administered to all participants by certified clinical psychologist.

### 2.2 General procedure

Upon the participants' first visit, an evaluation of their demographic and clinical characteristics was conducted, followed by psychological assessments and EEGs. Following this, participants were instructed on the proper usage of VR devices and how to operate VR handles to engage with the virtual environment. Subsequent to initial training, participants engaged in practice sessions for the CPT, with the trainers providing verbal explanations until a thorough understanding of the program's operation was achieved. Each participant was provided with a VR device containing the “Pay Attention!” program and instructed to complete only 1–2 blocks daily at home, adhering to the specified order of presentation, to mitigate performance deterioration due to fatigue. They were instructed to perform the program in a quiet room with no one around, however, the specific time and location for program execution were not restricted, allowing for the establishment of a natural evaluation environment. The CPT comprised 12 blocks over the ensuing 2 weeks. In case any issues with the device or questions arose during the performance, participants were instructed to contact the research team at any time. Following the 2-week period, at the second visit, the same psychological assessments and EEGs were repeatedly administered. Usability measures for the CPT were also collected.

### 2.3 Virtual reality integrated CPT

#### 2.3.1 VR-based CPT

Implemented into the virtual environment, the CPT appeared on a laptop monitor at the center of the user's field of view which the participants were instructed to focus on. A series of single letters or figures were presented at the middle of the laptop screen. The participants were instructed to respond by clicking the controller button for every stimulus except the one designated at the beginning of the task, requiring inhibition control (Conner's CPT-II, CCPT-II) (37). A single letter was shown randomly from a set of stimuli and inter-stimulus intervals varied across trials. The entire program comprised four conditions which differed in difficulty level, ranging from “low” to “very high”, characterized by distinct virtual environments (room, library, outdoor, and café). Each condition was split into three 15-min blocks during which 359 stimuli were presented. As such, 12 blocks were administered in total. While performing the CPT, various distractions were presented intermittently, characterized by their visual, auditory, mixed visual, and auditory natures, similar to what one might encounter in each situation. These distractions appeared either as persistent background elements (persisting throughout the block) or transiently (coming and going during the block for durations ranging from 3 s to 20 s), mimicking real-life circumstances.

The four graded difficulty levels and their corresponding virtual environments were as follows: (1) low (in front of the desk in a room at home), (2) medium (at the library), (3) high (outdoors, e.g., parks and streets), and (4) very high (at a café). A detailed description for each condition of difficulty level and the characteristics of the four virtual environments are presented in Table 1. The level of task difficulty was determined using two elements of the task: (1) the complexity of the stimuli presented as targets or non-targets in the CPT and (2) the total level of distraction. The set of stimuli used in the CPT comprised single letters at low and medium levels, while relatively complex figures were used at high and very high levels (Table 2). Regarding the level of distraction, although different types of distracting events occurred in each condition, the total level of distraction increased with the level of difficulty. To verify whether the levels of distraction were well graded, we quantified each distracting stimulus by counting the frequency and scoring the duration and intensity based on criteria established by our research team (Supplementary Table 1). We calculated the sum of the duration and intensity scores for each block. The total distraction level was calculated and extracted on a block-by-block basis using the following formula: the sum of the salience scores [(score of duration) × (score of intensity)] for each distracting stimulus. The total distraction level for each condition of difficulty level was the sum of the salience scores for subordinated three blocks. The scoring results for each block and condition are listed in Table 1. The total frequency of distractions, sum of intensity and duration scores, and total distraction levels increased with each difficulty level.

**Table 1:** The scores of indices reflecting distraction magnitude (duration, intensity, distraction level and frequency) in each block and condition.

**Task difficulty**	**Block**	**Sum of duration scores**	**Sum of intensity scores**	**Total distraction level (block unit)**	**Total distraction level (condition unit)**	**Total frequency of distractors**
Low	Block 1	3	1	3	48	22
	Block 2	15	10	15		
	Block 3	22	15	30		
Medium	Block 1	3	1	3	97	26
	Block 2	19	20	36		
	Block 3	23	2	58		
High	Block 1	19	13	22	112	33
	Block 2	23	19	40		
	Block 3	18	26	50		
Very High	Block 1	16	13	19	129	41
	Block 2	24	22	48		
	Block 3	28	41	62		

**Table 2:** Description of the CPT by each level, along with Images of set of stimuli and screenshots.

**Level (Location)**	**Set of stimuli in CPT**	**Description of distracting Stimuli**	**Screenshots**
Low (Room)	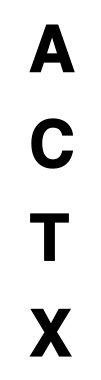	Family members are heard conversing outside of the room. The TV is on with loud music and speech, along with loud voices of family members.	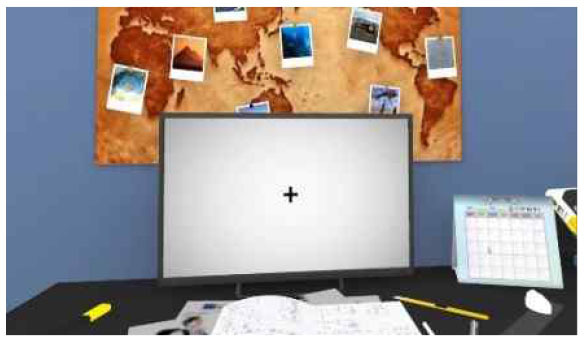
Medium (library)	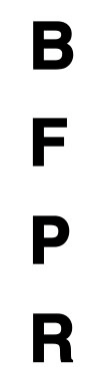	At a desk shared by multiple personnel, with intermittent auditory distractors through the silent background (e.g., the sound of pencil on paper, whispered speech, etc.). In the library's cafeteria with loud background noise and mixed visual and auditory distractors like an actual lunchtime (e.g., clearly heard conversations, background mumbling of people, sound of eating noodles, etc.).	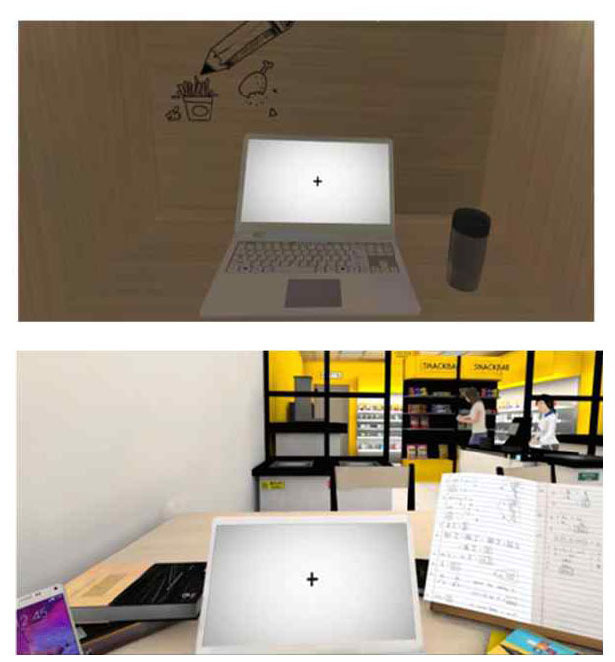
High (Outdoors)	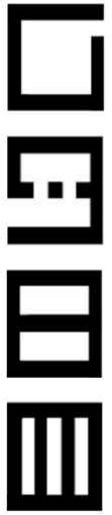	At the park with a passerby walking up and down, exercising far in the distance. In front of the school gate, amidst intermittent street noise and visual distractors such as cars driving and passers-by were presented. In the middle of the street, loud music is playing, and the road is packed with endless lines of automobiles. A friend abruptly talks to the user.	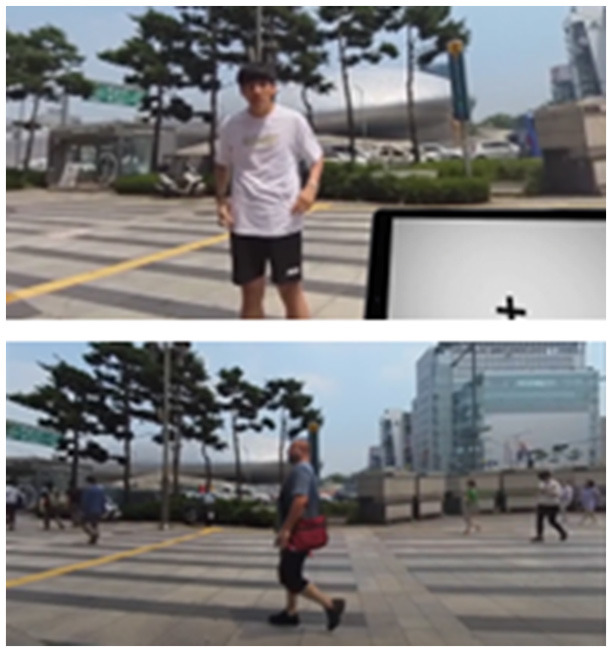
Very high (Café)	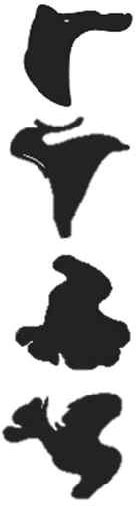	In a café, there are only two or three people in the café and the chairs are mostly empty. More people are in sight with more words heard, along with other visual and auditory distractors (the employee approaching with the ordered menu, the noise of the blender, etc.). A friend is sitting in front of the user making variant movement, and most chairs are full in the background. The friend talks to the user once during the task. The sounds of ordering, drink preparation, etc., are much louder.	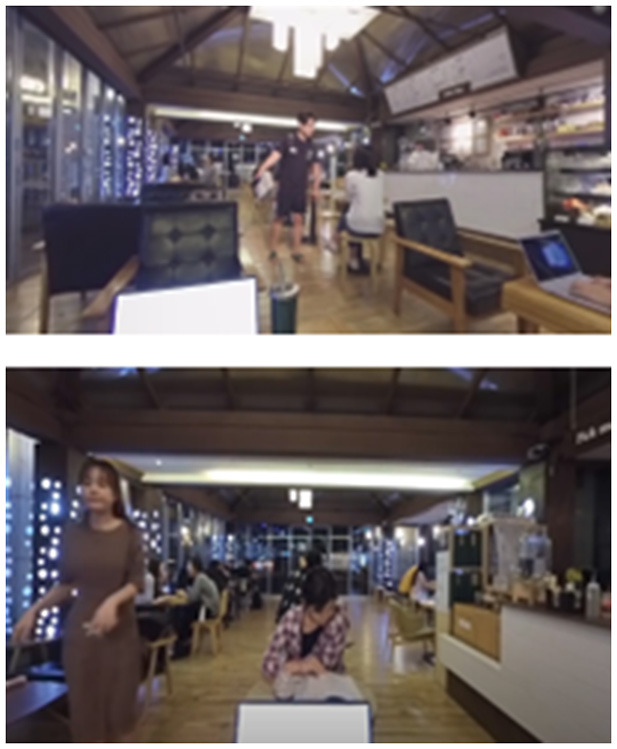

To assess CPT performance, the five standard CPT attention variables [accuracy (AC), commission error (CE), omission error (OE), mean reaction time (RT), and reaction time variability (RTV)] of the participants were extracted and calculated for each block. AC represents the rate of correct hits. OEs occur when participants fail to respond to target letters (every letter except the designated one) while CEs are made when responses are given to non-targets (the designated stimulus). These three variables were recorded as frequencies, and they indicate the correctness of the overall performance. In particular, OEs are considered a marker of inattention, whereas CEs are thought to reflect impulsivity (38). RT denotes the average speed of correct responses, and RTV was defined as the standard deviation of reaction times toward correct hit trials divided by the mean reaction time (39, 40). RT and RTV are considered as measures of vigilance. Especially, RTV reflects lapses in attention lead to temporary slowing of responses and variable reaction times (40).

#### 2.3.2 Virtual environment

A VR system was developed using a mobile platform. The content was designed on Unity 2018.3.11f1 software (Unity Technologies, San Francisco, CA). The VR program was implemented on a Samsung Galaxy 8+ smartphone equipped with a Samsung Gear VR device (Samsung Electronics). The program was presented via Head Mounted Display (HMD), which has a 360-degree field of view. The avatars and structures present in the virtual environments at low (room) and medium (library) levels were built using 3Ds Max 2014. The virtual environments for the other two levels were created as video recordings of the actors, captured using an Insta360 Pro camera. Surroundings of all four conditions, either virtually programmed or recorded through the camera, underwent spatial mapping through positional tracking. Any alteration in the participants' head position in the real world led to a corresponding adjustment in the position of the HMD in the virtual environment.

### 2.4 Measurement

#### 2.4.1 Electroencephalograms

We used EEGs taken before and after the study to gain further insights into the possible neural mechanisms related to distractibility in healthy controls. Although the theta-beta ratio (TBR), referred to as the ratio between absolute theta power (4–8 Hz MeanSq) and beta-1 power (13–21 Hz MeanSq), is known to be inversely related to attentional control (41), there have been inconsistent reports on whether TBR changes when performing cognitive tasks in controls without clinically significant attention deficits (42, 43). In addition, in the VSR program developed by Wiebe et al. (4), no significant associations between attention and TBR changes were observed. Since some studies have reported that a decrease in the TBR of controls without ADHD was modulated by task difficulty, with reductions more pronounced in more challenging tasks (44). As we have made efforts to design the program to be more cognitively demanding than existing VR-based CPTs, we expected to obtain meaningful results in TBR. Although the ideal approach involves monitoring TBR changes concurrently during the program, the integration of EEG and VR can potentially introduce artifacts that interfere with the signals. For example, placing an HMD on top of an EEG cap may exert pressure on the electrodes, and head movements may result in an increased occurrence of motion artifacts (4, 45). Therefore, EEGs were measured both before and after the program. Data were collected using the ProComp Infiniti System with Biograph Infiniti Software (T7500M, Thought Technology, Montreal, Canada) on a Hewlett Packard laptop. ProComp Infiniti is an eight-channel multimodality encoder, for which we used a single channel appropriate for obtaining theta and beta-1 brainwaves. TBRs were calculated to objectively assess the participants' attention levels (46, 47).

#### 2.4.2 Psychological confounding factors

We assessed the participants for possible psychological symptoms that may affect their CPT performance, including depression, anxiety, and stress. Depressive symptoms were assessed using the Korean version of the Depressive Symptomatology (KIDS-SR) (48). Each variable of the 30-item questionnaire was scored on a scale of 0 to 3. Anxiety levels were assessed using the State-Trait Anxiety Inventory (STAI), Korean version, with variable values ranging from 0 to 3 (49). The Perceived Stress Scale (PSS) was used to assess the degree to which situations in participants' lives were perceived as stressful, with variable values ranging from 0 to 4 (50). Symptom scales were compared before and after the study, and correlations between the scales and attention variables were analyzed.

#### 2.4.3 Usability measures

Four self-report questionnaires assessing the usability of the VR program were administered at the second visit after the two-week study period. To assess the presence level, the psychological state of “being there” mediated by the VR experience, the Presence Questionnaire (PQ) version 3.0 was used. The original PQ consists of 29 items rated on a 7-point Likert scale. Cronbach's alpha for the PQ was reported to be 0.57–0.89 (51). The score ranges from 29 to 203 and can be graded as 0–67 as low, 68–133 as medium, and high (> 133). In this study, 22 items related to adaptation, involvement, and interface quality were included. The remaining seven items that were not suitable for the program, such as factors of touch and the ability to move or manipulate objects, were excluded. Therefore, the modified total score range was 22–154. A Simulator Sickness Questionnaire (SSQ) was used to assess simulator sickness experienced during the test (52). Variable values were rated from 0 to 3 and subscales representing nausea, oculomotor disturbances, and disorientation were calculated. A higher score indicates a higher level of VR-induced sickness. The System Usability Scale (SUS) was used to evaluate usability as perceived by the user (53). The SUS is a reliable, quick, and easy method that provides a single score on a scale that is easily understood by a wide range of people. The SUS is composed of 10 statements related to various aspects of usability that are scored on a 5-point scale of strength of agreement. The final SUS scores ranged from 0 to 100, with higher scores indicating higher perceived usability. We also assessed the users' perceived satisfaction with the VR system using a modified 17-question version of the Post-Study System Usability Questionnaire (PSSUQ) with variable values rated from 1 to 7 (54). Three statements, each addressing whether the user could effectively, quickly, or efficiently complete the tasks and scenarios, were combined into one statement: “I was able to complete the tasks and scenarios quickly using this system.” The total score was calculated as the average of the individual scores, and when possible, the subscales were calculated and analyzed.

### 2.5 Statistical analysis

As our sample size was smaller than 30, which is considered the standard for parametric tests according to the central limit theorem, we conducted additional tests for data normality, including skewness and kurtosis tests, and visually inspected the Q-Q plots. We performed repeated measures ANOVA for each attention variables with within-subject factors of “task difficulty” (4: Low vs. Medium vs. High vs. Very high). Because the sphericity assumption was not satisfied, the Greenhouse-Geisser correction was applied. For reporting ANOVA effect sizes, partial eta squared (η^2^*)* was used. According to Cohen (55), η^2^ = 0.01 indicates a small effect, η^2^ = 0.06 a medium effect, and η^2^ = 0.14 a large effect. Additionally, to examine the effect of distracting stimuli on CPT performance, we calculated rank correlation coefficients using Kendall's tau (τ) between the four indices reflecting distraction magnitude of each block (frequency, sum of intensity scores, sum of duration scores, and total distraction level) and five attention variables. We also analyzed the correlations between psychological confounding factors and attention variables. Wilcoxon signed rank-sum tests were conducted to compare the EEG TBR and self-reported questionnaire results before and after the program. Statistical analysis was performed using SPSS version 26.0.0.2 and all results were considered statistically significant at *p* < 0.05.

### 2.6 Ethics approval

This study was approved by the Institutional Review Board of Yonsei University College of Medicine, Gangnam Severance Hospital, and informed consent was obtained from all participants.

## 3 Results

### 3.1 Demographic characteristics

In total, 20 adults, including 10 men and 10 women, participated in the study with an age range of 22 to 52 years (mean 28.3 years, SD 6.5 years). According to the logs collected from the participants' VR equipment, the participants performed 1–6 blocks per day (mean = 1.83, SD = 1.34), and the total number of days spent completing the program ranged from to 2–12 days (mean = 6.66, SD = 2.65). Psychological confounding factors, including depressive symptoms, anxiety, and stress evaluated at baseline, were all within the normal range (Table 3).

**Table 3:** Demographics and clinical characteristics.

	**Mean (SD)**	**Range**
**Demographics**		
Age (years)	28.3 (6.5)	22–52
Sessions per day	1.83 (1.34)	1–6
Total days spent completing program	6.66 (2.65)	2–12
**Psychological confounding factors (pre-test)**		
KIDS-R	5.1 (4.53)	0–18
STAI	70.0 (18.03)	43–124
PSS	13.8 (5.61)	4–25

KIDS-SR, Korean version of the Inventory of Depressive Symptomatology-Self-Report; STAI, State-Trait Anxiety Inventory; PSS, Perceived Stress Scale. N = 20.

### 3.2 VR-based CPT performance

**Figure 1:**
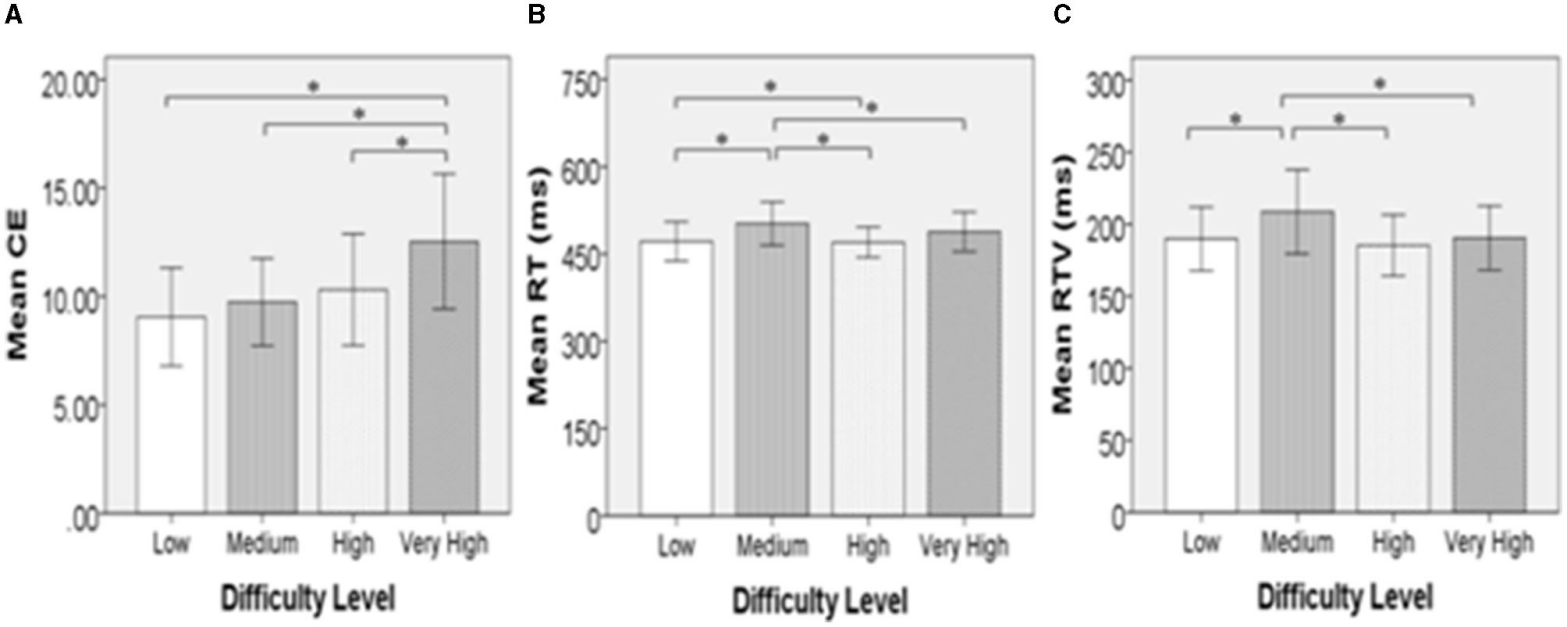
Graphs indicating comparison between four locations. Standard deviations (±2SD) are represented as error bars. **(A)** Shows that the difference in mean frequency of commission errors was significance in all pairs with café condition. **(B)** Indicates that the library condition had a significantly delayed mean reaction time compared to the room, outdoors, and café. **(C)** Shows that the mean reaction time variability was significantly larger in the library than in the room or café. CE, commission error (frequency); RT, averaged reaction time (ms); RTV, reaction time variability (ms). ^*^p < 0.05.

Two participants were excluded from the statistical analyses because attention variables were not properly recorded owing to technical problems. The repeated-measures ANOVA showed that difficulty level had a significant effect on CE [F (2.080, 17) = 4.220, *p* < 0.05], RT [F (1.769, 17) = 3.507, *p* < 0.05], and RTV [F (2.125, 17) = 4.281, *p* < 0.05] (Table 4). There were significantly more frequent OEs in the “very high” condition, in which the most complex CPT stimuli and the highest level of distraction were presented, than in the other conditions (Figure 1). While not all pairs of conditions were statistically significant, we found that the means of CEs for the four conditions increased with the difficulty of each level: low (M = 9.06, SE = 1.13) < medium (M = 9.741, SE = 1.01) < high (M = 10.32, SE = 1.29) < very high (M = 12.54, SE = 1.56) (Figure 1). Descriptive statistics, including mean and standardized errors, are presented in Table 4. Meanwhile, the “medium” condition exhibited significantly longer RTs and greater RTV when compared to the other conditions (Figure 1).

**Table 4:** Statistical results of repeated measures ANOVA for attention variables.

**Attention variable**	**Difficulty level**				**Adjusted *df***	***F***	***p***	***η2***
	**Low**	**Medium**	**High**	**Very high**				
	**Mean (** ***SE)***							
AC	95.332 (0.626)	95.476 (0.656)	95.409 (1.197)	95.997 (0.560)	1.664, 17	0.321	0.688	0.019
OE	7.944 (3.351)	6.556 (1.690)	4.704 (1.122)	4.796 (1.242)	1.410, 17	0.982	0.361	0.055
CE	9.056 (1.130)	9.741 (1.006)	10.315 (1.287)	12.537 (1.560)	2.080, 17	4.220	**0.022** ^*^	0.199
RT	471.588 (8.244)	502.217 (18.638)	470.171 (13.074)	488.117 (17.020)	1.769, 17	3.507	**0.048** ^*^	0.171
RTV	189.829 (11.062)	208.751 (14.616)	185.406 (10.578)	190.488 (11.170)	2.125, 17	4.281	**0.020** ^*^	0.201

Effect of difficulty level of task was significant in three variables of CE, RT, and RTV.

AC accuracy (%), CE commission errors (frequency), OE omission errors (frequency), RT mean reaction time (ms), RTV reaction time variability (ms).

* p < 0.05. SE = Standardized Error, N = 18. Significant *p*-values have been bolded.

As a result of rank order correlation analysis of Kendall's tau, a significant correlation was observed between the scores of total distraction level in each block and accuracy in CPT (τ = −0.443, *p* < 0.05). The sum of intensity scores for each block was significantly correlated with CEs (τ = 0.543, *p* < 0.05). The coefficients of the two significant correlations were moderate (Table 5).

**Table 5:** Statistical results of rank correlation coefficients of Kendall's tau-b.

	**AC**	**OE**	**CE**	**RT**	**RTV**
	**tau-b (**τ**)**				
Sum score of intensity	−0.308	0.092	**0.543** ^*^	0.031	0.185
Sum score of duration	−0.264	0.202	0.344	0.233	0.264
Score of total distraction level	**−0.443** ^*^	0.321	0.390	0.229	0.321
Total frequency of distractors	−0.291	0.323	0.309	0.259	0.291

Significant negative correlations were observed between the scores of total distraction level and AC. Positive correlation was significant between the sum of intensity scores and CE.

AC accuracy (%), CE commission errors (frequency), OE omission errors (frequency), RT mean reaction time (ms), RTV reaction time variability (ms). N = 12.

* p < 0.05. Significant *p*-values have been bolded.

Psychological confounding factors that may affect attention variables in the CPT were assessed before and after the study. There were no statistically significant differences in depressive symptoms, anxiety, or stress before and after the study (*p* = 0.47, 0.92, and 0.85, respectively; Supplementary Table 2). In addition, there were no correlations between the attention variables and the differences in measures obtained from the questionnaire assessments before and after the study (Supplementary Table 3).

### 3.3 Change of electroencephalograms

Two participants' EEGs with their eyes closed were not assessed before and their data were therefore excluded from the analysis. TBR before and after the study did not show a significant difference (Table 6).

**Table 6:** Theta/beta ratios pre-/post- program.

	**Pre-test**		**Post-test**		***p*-value**
	**Mean**	**SD**	**Mean**	**SD**	
Eyes open	2.49^a^	0.99	2.66^a^	1.18	0.550^a^
Eyes closed	2.27^b^	0.93	2.38^a^	1.16	0.948^b^

a n = 20.

b n = 18.

### 3.4 Usability measures

Although 7 items were excluded, the mean value of total PQ scores was 97, with a standard deviation of 18, showing a medium level based on the original full version (0–67:low, 68–133:medium, ≥134:high). Scores for VR sickness (SSQ) were quite high, with a mean of 64.9 and a standard deviation of 34.6. Subscales representing nausea, oculomotor disturbance, and disorientation showed means of 19.6, 72.8, and 79.3, respectively. SUS scales showed a mean of 66.3 with a standard deviation of 14.2. This mean score stands between “fair” and “good” according to a study performed by Lewis (53). The PSSUQ showed a total score of 5.0 with a standard deviation of 1.0. Subscales representing system usability, information quality, and interface quality had means of 5.4, 5.0. 4.4, respectively.

In addition, we collected short-answer feedback from the participants during a debriefing session after completing the program. The participants complained that the avatars created by 3-dimensional modeling in the library setting were eerie and scary. They also pointed out that it was difficult to maintain a focused perspective throughout the sessions.

## 4 Discussion

In this study, we demonstrated the potential of a newly developed VR-based CPT as an ecologically valid assessment tool with diverse difficulty levels. Our sample of 20 non-clinical participants attending the current study completed the over 2-week period program without giving up. This is particularly significant and encouraging, as it demonstrates the utilization of this VR program as a home-based assessment tool in natural settings. The mean PQ scores were rated as medium even when based on the original version, and the SUS mean score was between “fair” and “good”. Moreover, the total and subscale scores of the PSSUQ for our VR program were above average. These results strongly support the acceptability and usability of the program. One of the main focuses of our study was to collect ecologically valid data representing the participants' natural performance with voluntary motivation in a home-based setting, rather than standardized collection within a controlled environment. While all participants completed the program as intended without supervision, there were differences in the frequency of usage among the participants. However, because the quality of the CPT data obtained was generally acceptable, these variations were considered natural responses. Among the usability measures, the level of cybersickness was quite high with a large standard deviation, suggesting that our program may have caused discomfort to some participants. As some participants completed the program in only 2–3 days, using it for over 60 min per day, excessive use of the VR system is likely to have induced symptoms of cybersickness (56). Therefore, minimum control and guidelines for usage hours may be required to prevent and manage discomfort during the program. Ultimately, technical improvements and solutions to minimize cybersickness are important challenges that must be overcome.

The key point of our study was that the adjustment and segmentation of difficulty levels in the VR-based CPT were achieved, and this effort was validated by examining the impact of different levels of difficulty on attention variables. Significant differences in CPT performance emerged based on the task difficulty. For example, especially in the “very high” condition, which was designed to present the highest level of distraction and the most complex CPT stimuli, the performance accuracy predominantly decreased, resulting in more frequent CEs (i.e., pressing the key when a non-target stimulus appeared) compared to the other conditions. Furthermore, the average frequency of CEs exhibited a consistent ranking based on the four difficulty levels.

Although the difficulty level of each condition was designed and determined based on the complexity of target and non-target CPT stimuli as well as the distraction level, we also found a significant impact of distraction itself on CPT performance in the results of our correlation analysis. A noticeable trend was observed: as the total distraction level in each block increased, the performance in the CPT declined, indicated by a decrease in accuracy. In addition, there was a trend toward an increase in CEs with the sum of the intensity of the distracting stimuli in each block. These results imply that distracting environmental stimuli can induce errors in impulsivity control and reduce processing accuracy even in individuals without clinically significant attention deficits. These findings emphasize the importance of considering distraction as a crucial factor in CPT performance, as it makes participants perceive the task as more difficult, imposing an additional cognitive burden independent of the inherent task demands. This suggests that managing and minimizing distraction is essential for maintaining accuracy and attention during cognitive tasks such as the CPT.

Previous studies have reported mixed results regarding ubiquitous distractibility in healthy controls. Some researchers have failed to reliably measure CPT performance owing to ceiling effects (4, 21, 29). By diversifying the difficulty level of the CPT, our study detected the impact of distraction on attentional processing and underperformance in adults without ADHD. Whether distractibility is more closely related to commission or omission errors, reflecting inattentiveness, has been controversial (5). This issue has been treated as important for understanding how neuropsychological correlates of distractibility in the CPT manifest in different ADHD subtypes (57–59). The current study highlights the ubiquity of distractibility in the control group and suggests that it may be associated with impulsivity rather than inattention. Investigating individuals with ADHD in light of this notion could offer valuable insights for future investigations.

Meanwhile, significantly prolonged mean RTs and the highest RTVs, which are known to reflect both low processing efficiency and heightened processing demand, were observed in the “medium” level condition. Since the total distraction was at a moderate level, and the set of stimuli presented in the CPT consisted of relatively simple letters at this level, the results were contrary to our expectations. Given the short-answer feedback we received from the participants during the debriefing, stating that the avatars created by 3-dimensional modeling at the medium level were eerie and scary, this perceptual discomforting experience might have had a negative influence on CPT performance. This phenomenon is known as the Uncanny Valley effect (i.e., people feeling unpleasant about human-like non-human characters), which can be exacerbated when using a head-mounted display compared to a computer monitor (60–62). This effect can be explained with the categorical perception hypothesis, which assumes that every time we are exposed to a face-like stimulus, we automatically ask ourselves whether the face represents a human or a non-human being (63). According to this hypothesis, an Uncanny Valley is induced by the difficulty in categorizing agents with mixed human and nonhuman features, which is associated with cognitive conflict and increased cognitive load (64). Although we did not directly evaluate the Uncanny Valley effect in our study, there is a possibility that the processing demands induced by human-like avatars at the “medium” level increased, potentially leading to a decrease in the efficiency of CPT performance. The video capture method used for the “high” and “very high” levels, which involved taking video shoots with actors at an actual real-world location, received better feedback that the setting was more realistic and less awkward. Therefore, replacing 3D avatars with video-capture recordings of real humans and repeated verification may be desirable for revised and advanced versions of our program.

We intended to evaluate the TBR EEGs parameter, which is known to have a negative correlation with sustained attention and attentional control (46). This is incongruent with our study, in which there was no statistically significant change in the TBR after CPT performance. To our knowledge, while there are a considerable number of reports on TBR, theta power, or beta power as discriminating factors between individuals with and without ADHD (47, 65, 66), previous literature on how much TBR is influenced when those without ADHD are distracted is limited (42). Recent studies have reported that cognitive task difficulty is associated with TBR (44); however, the current study found no significant decrease despite applying various levels of CPT difficulty. It is possible that some changes were not detected because TBR changes were not inspected during the CPT simultaneously. We chose not to do simultaneous assessment as it would have limited home-based application of the test and to prevent motion artifacts and noise signals that may be induced by the HMD device. Therefore, it is necessary to devise a method for simultaneous multimodal measurements, including EEGs, to sensitively detect neural markers. Because we identified some significant results in behavioral parameters in our study, continuous efforts to discover the neural mechanisms of distractibility and attention control that may represent CPT performance in controls, might be meaningful. Further investigation is needed to determine whether the TBR is a sensitive marker of attention in controls.

Overall, our effort to enhance the diagnostic utility of CPT as an assessment tool by diversifying the difficulty level into various grades is a useful solution for eliciting individuals' attention problems more effectively in an ecologically valid environment. This allowed us to observe how healthy adults reacted to different task difficulties in close-to-real-life environments. Furthermore, we identified a controlled stimulus environment in which attention challenges could be presented along with the precise delivery and control of “distracting” auditory and visual stimuli within our VR environment. Understanding how distractors affect attention is crucial for shaping future research and cognitive assessment/training programs for both typically functioning individuals and those facing attention-related challenges. We believe that the implications of the current study provide direction for deepening the comprehension of typically functioning adults and could serve as a steppingstone for applying our program and investigating attention impairment in individuals with ADHD in the future. Notably, the use of this experimental control in a home-based environment maximized its ecological validity. This high level of experimental control enhanced the attention assessment tasks, benefiting both their practical utility and psychometric utility. Our VR-based CPT facilitates the provision of more personalized applications to users, offering a range of task difficulty levels, and improved accessibility and convenience.

Although the findings are encouraging, an important limitation is that our classification criteria for scoring total distraction in each block were not based on references, but rather on our researchers' discretion. Despite efforts to enhance inter-rater reliability through discussion among the three researchers, there was a lack of objective measurements of distracting stimuli. If the distractors were quantified in a more standardized manner, further analyses could have been performed. For instance, assessing how different components of distraction provided by various VR environments influence attention variables, with auditory distractors potentially represented by intensity (e.g. decibels) and visual distractors by quantified virtual proximity or the proportion of the participant's field of view. Meanwhile, the EEGs were measured only twice, before and after the study; therefore, null findings in EEGs should be interpreted with caution. The decision to measure EEGs separately was necessitated by the physical difficulty of wearing several devices at home during the administration of “Pay Attention!” program. Simultaneous measurements within the program would be beneficial for collecting more plausible data that reflect the effect of distractibility in an advanced version. Additionally, we did not examine the concurrent validity of our program with other established assessments, such as traditional CPTs or psychological questionnaires evaluating attention ability. Given that numerous current CPT programs have not successfully established meaningful correlations between CPT performance and other measurements, or self-reports indicative of impairment in daily life, it is crucial to conduct repeated testing and ensure this aspect during the preliminary stage for reliable utilization. Moreover, concerning the environmental conditions in our home-based program, there were no specific constraints on the time and location of program execution, except for the requirement of a quiet room with no other individuals present. While this approach aimed to create ecologically valid surroundings for the assessment, it also introduced potential variability in external factors such as noise and lighting. Despite our efforts to conduct multiple sessions to mitigate these inconsistencies, there is a possibility that they may have influenced attentional performance and induced differences in experimental conditions between participants. In future studies, comparing outcomes in both home and laboratory settings for consistency would be beneficial. Additionally, incorporating environmental monitoring tools could provide another valuable option for quantifying the impact of these variables on attention. Lastly, technical problems with the program and participants reporting a high level of cybersickness were noted. At a more granular level, it is plausible that the Uncanny Valley effect influenced CPT performance. Related risk factors should be considered, and adequate supervision and guidelines should be provided to users when conducting the program. Technical improvements would be beneficial for creating a more perceptually comfortable and safer virtual environment. After rectification and advancement, the next step would be to identify the convergent validity of our program by comparing it with other established versions of CPT program and apply it to a wider range of groups including those with ADHD and other attention problems.

## 5 Conclusion

Our study demonstrated that the newly developed VR-based CPT program designed to closely mimic various real-life environments, with a focus on adjusting and diversifying task difficulty levels, was acceptable and feasible. The main result was that significant differences in CPT performance emerged based on task difficulty. This result provides a useful solution for more effectively eliciting attention problems in an ecologically valid environment. We also found a significant impact of distraction on CPT performance, implying the importance of considering distraction as a crucial factor in CPT performance. The high level of experimental control in our program enhanced the attention assessment tasks, benefiting both their practical utility and psychometric properties. Although the VR-based CPT exhibited a high degree of user immersion and system usability, VR-induced sickness has emerged as a significant concern. As we continue to advance in the realm of VR technology, addressing usability issues is imperative to create a more comfortable and perceptually appealing environment for users, thereby optimizing the utility of VR programs.

## Data availability statement

The raw data supporting the conclusions of this article will be made available by the authors, without undue reservation.

## Ethics statement

The studies involving humans were approved by the Institutional Review Board of Yonsei University College of Medicine, Gangnam Severance Hospital. The studies were conducted in accordance with the local legislation and institutional requirements. The participants provided their written informed consent to participate in this study.

## Author contributions

H-KO: Data curation, Formal analysis, Methodology, Writing–original draft. YC: Data curation, Formal analysis, Methodology, Writing–original draft. J-JK: Funding acquisition, Project administration, Writing–review & editing. BS: Data curation, Formal analysis, Writing–review & editing. S-JK: Investigation, Writing–review & editing. SP: Investigation, Writing–review & editing. J-HS: Software, Supervision, Writing–review & editing. SK: Investigation, Validation, Writing–review & editing. EK: Project administration, Supervision, Writing–review & editing, Funding acquisition.
