# Supplementary material for: Advancing ecological validity and clinical utility in virtual reality-based continuous performance test: exploring the effects of task difficulty and environmental distractors
Source: Front Psychiatry. 2024 Jan 17;14:1329221. doi: 10.3389/fpsyt.2023.1329221 (PMC10832060; doi:10.3389/fpsyt.2023.1329221)
Supplement: Supplementary file 1 [file Table_1.DOCX]

Supplementary Material

# Supplementary Figures and Tables

## Supplementary Tables

Supplementary Table 1. The scoring criteria of distracting stimuli.

|  | | Score | Criteria |
| --- | --- | --- | --- |
| Duration | | 0 | No distractors |
|  |  | 1 | Less than 10 seconds |
|  |  | 2 | More than 10 seconds but less than 20 seconds |
|  |  | 3 | Longer than 20 seconds |
| Intensity | Auditory Stimuli | 0 | No sound or noise. |
|  |  | 1 | Small and faint sounds in the distance (e.g. whispering, mumuring). |
|  |  | 2 | Clear and identifiable sound (e.g. music, the sound of a mixer, talking, desk dragging). |
|  |  | 3 | Loud noise, music, or din. Sounds from a close distance (e.g. A passerby talks to the user abruptly). |
|  | Visual Stimuli | 0 | No distractors |
|  |  | 1 | Fixed distractors (e.g. clutter) or moving distractors are visible in the distance. |
|  |  | 2 | An identifiable moving distractor exists nearby |
|  |  | 3 | Multiple distractors are continuously moving or approaching closely |

**Supplementary Table 2. Effect of training on psychological confounding factors**

|  | Pretest | | Posttest | |  |  |
| --- | --- | --- | --- | --- | --- | --- |
|  | Mean | SD | Mean | SD | *p*-value | |
| KIDS-SR | 5.1 | 4.5 | 4.1 | 4.1 | 0.109 | |
| STAI | 70 | 18 | 69.5 | 16 | 0.537 | |
| PSS | 13.7 | 5.6 | 14.1 | 4.7 | 0.795 | |

*Note.* KIDS-SR- Korean version of the Inventory of Depressive Symptomatology-Self-Report, STAI- State-Trait Anxiety Inventory, PSS-Perceived Stress Scale. N = 20.

**Supplementary Table 3. Effect of psychological confounding factors on training**

|  | Mean change (SD) | p-value | | | | |
| --- | --- | --- | --- | --- | --- | --- |
|  |  | AC | CE | OE | RT | RTV |
| KIDS-SR | -1.00 (3.33) | 0.676 | 0.804 | 0.846 | 0.878 | 0.660 |
| STAI | -0.28 (8.74) | 0.595 | 0.851 | 0.835 | 0.415 | 0.544 |
| PSS | -0.28 (4.11) | 0.488 | 0.628 | 0.889 | 0.844 | 0.918 |

*Note.* AC accuracy (%), CE commission errors(frequency), OE omission errors(frequency), RT mean reaction time(ms), RTV reaction time variability(ms). N = 18.
